# Temperature and energy effects on secondary electron emission from SiC ceramics induced by Xe^17+^ ions

**DOI:** 10.1038/s41598-017-06891-9

**Published:** 2017-07-25

**Authors:** Lixia Zeng, Xianming Zhou, Rui Cheng, Xing Wang, Jieru Ren, Yu Lei, Lidong Ma, Yongtao Zhao, Xiaoan Zhang, Zhongfeng Xu

**Affiliations:** 1 Institute of Science and Technology for Laser and Particle Beams, Xi’an Jiaotong University, Xi’an, 710049 China; 2 School of Science, Xi’an Jiaotong University, Xi’an, 710049 China; 3 Institute of Modern Physics, Chinese Academy of Science, Lanzhou, 730000 China; 4 Ion Beam and Optical Physical Laboratory, Xianyang Normal University, Xianyang, 712000 China

## Abstract

Secondary electron emission yield from the surface of SiC ceramics induced by Xe^17+^ ions has been measured as a function of target temperature and incident energy. In the temperature range of 463–659 K, the total yield gradually decreases with increasing target temperature. The decrease is about 57% for 3.2 MeV Xe^17+^ impact, and about 62% for 4.0 MeV Xe^17+^ impact, which is much larger than the decrease observed previously for ion impact at low charged states. The yield dependence on the temperature is discussed in terms of work function, because both kinetic electron emission and potential electron emission are influenced by work function. In addition, our experimental data show that the total electron yield gradually increases with the kinetic energy of projectile, when the target is at a constant temperature higher than room temperature. This result can be explained by electronic stopping power which plays an important role in kinetic electron emission.

## Introduction

The interaction of intense radiation and charged particles with solid targets has drawn considerable interest, not only from fundamental physics, but also from many applications such as material modifications, X-rays source devices, radiation physics, chemistry, biology, plasma-wall interactions, and surface analysis as well^1–11^. Electron emission from solid surfaces under bombardment by charged particles is a well-known emission phenomenon, which is usually described by the mean number of emitted electrons per incident projectile, the electron emission yield *γ*. The knowledge of electron emission yield gives important information about the basic interaction mechanism between projectiles and solids and contributes to the understanding of impact phenomena like ion-track production^1–3^. It is well known that electron emission yield depends on the charge state, energy, atom number of the projectile and the angle of incidence and so on^8–11^. We have also done some work about the effects of recoiling atoms and charge state on electron emission yield *γ* ^12, 13^. Since the dependence of *γ*on target temperature is small, the temperature effect on electron emission had not been studied extensively until about ten years ago^14–17^. H. Hopman *et al*. reported an increase of 3% of the electron induced emission yield for Cu when a heated sample is cooled down by 300 K^14^. O. Benka *et al*. found the yield decreased slightly with increasing temperature for electrons, H^+^ and He^2+^ ions impacting on Al, Cu and Ag samples^15, 16^. A. Stacey *et al*. found that the electron emission yield from single crystal and polycrystalline diamond film surfaces increased with temperature in the 293–473 K range^17^. In previous studies, it was found that temperature effect on electron emission was not obvious, when projectiles with low charge state were employed. We have not found published data about the temperature effect on electron emission when the target was bombed by highly charged ions (HCIs). HCIs are efficient carriers of energy, due to their kinetic energy and potential energy, and due to the high ionization state as well. Secondary electron emission induced by HCIs is commonly ascribed to two different mechanisms, the potential emission process (PE) and the kinetic emission process (KE)^18, 19^.

In this work, we investigate the temperature effect on electron emission yield induced by Xe^17+^ ions in the range of 463–659 K. Silicon carbide (SiC) ceramic materials with potential application is selected as target. SiC ceramics is a strong covalent bond compounds, which is widely used in harsh conditions of industrial areas, such as high temperature carriers, atomic reactor structure materials, space engine combustion chamber. At present, a very important application of SiC is the wall material as a magnetic confinement fusion device. However, secondary electrons which are induced by *α* particles generated by nuclear fusion, will affect the high-temperature plasma in fusion device. Therefore, the study of electron emission from SiC ceramic has practical significance. Our results indicate that the temperature effect on electron emission is more obvious than reported by others’ results^14–17^. That is discussed by analyzing the temperature effect on the work function. Meanwhile, the dependence of electron emission yield on the kinetic energy of projectile is studied at a constant target temperature, we have done some work about it at room temperature. Our result will be useful for the investigation of electron emission at temperatures above room temperature and the surface analysis of SiC ceramics.

## Experimental setup

**Figure 1:**
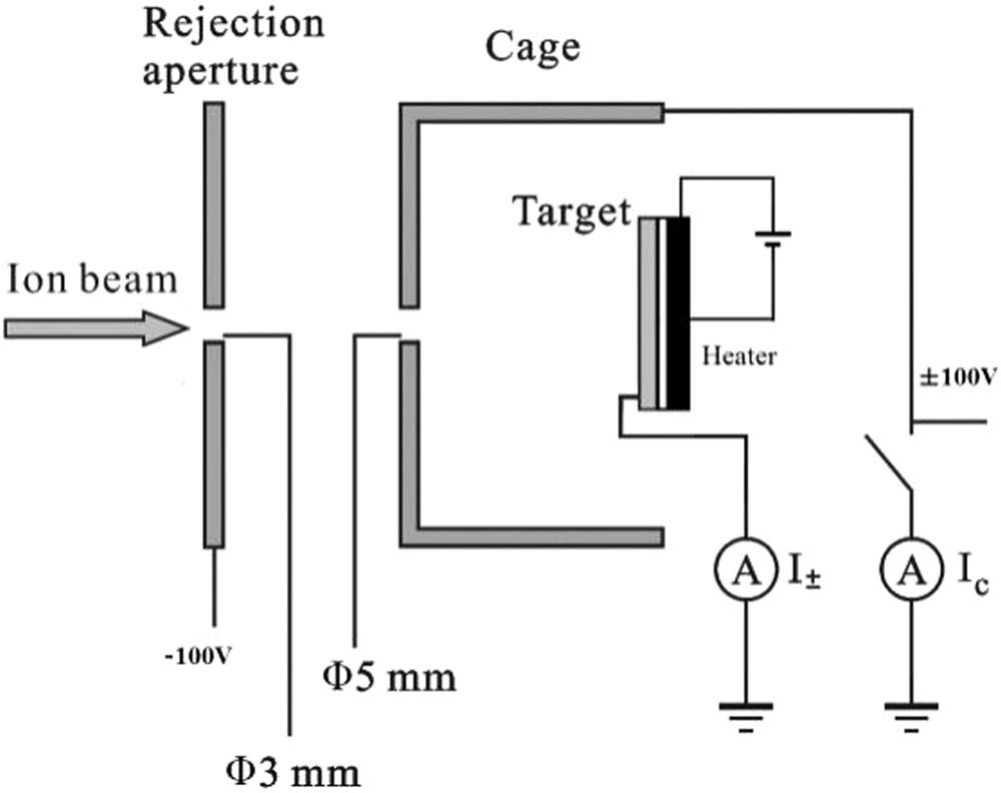
Schematic diagram of the experimental setup used for measuring total electron yield.

The experiment was performed with the 320 kV electron cyclotron resonance ion source (ECRIS) platform at the Institute of Modern Physics, Lanzhou^20^. A large number of experiments about interaction of highly charged particles with solid surfaces have been performed on this platform^11–13, 21–23^. The description of experimental method can be found elsewhere^11–13^. The experimental setup in our experiment is placed in the ultrahigh vacuum (UHV) target chamber (10^−9^ mbar). As shown in Fig. 1, it consists of four major parts, namely, an adjustable beam collimator, a rejection aperture with diameter of 3 mm, an UHV heater and a cage. The beam defining collimator serves to prevent the incoming ions from directly impacting the rejection aperture and has a diameter of 3 mm. A rejection electrode at a potential of −100 V is placed before the cage that prevents electrons from escaping and thus further enhances the electron collection efficiency. The cage is operated at a voltage of ±100 V and surrounds the target in order to collect or to suppress electrons emitted from the target. The cage has an aperture of Φ5 mm as an ion beam entrance, by which the incident ion beam is collimated before it is able to reach target surface. The collimation was performed by monitoring the current on the cage and the target. The best position was fixed when the current of cage equaled to zero, and the target current reached a peak. The samples were mechanically polished, washed using acetone and ethanol, and cleaned by heating in UHV. The temperature of target was controlled by an UHV heater made in HeatWave Labs. There was an insulation sheet placed between target and heater. Highly charged ion beams, with ion current in the range of 80–480 nA, were focused and collimated to a diameter of 3 mm. The current at the target was measured by a pico-ampere meter and a time integration constant of about 30 s was used.

The total electron yield is given as

1 $$\gamma =q\frac{{I}_{+}-{I}_{-}}{{I}_{-}}$$

where *q* is the charge state of the incident ion, *I*_+_ and *I*_−_ are target currents for ±100 V applied to the cage.

## Results and Discussion

**Figure 2:**
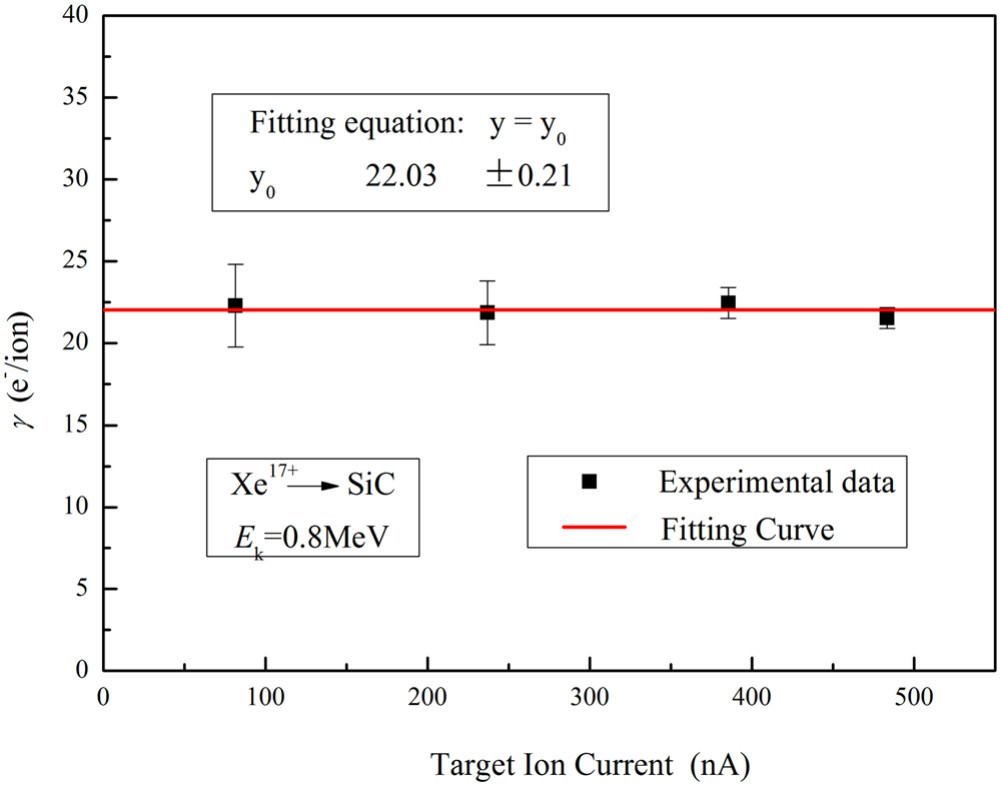
Total electron yield *γ* as a function of ion current for 0.8 MeV Xe^17+^ impacting on SiC ceramics at a temperature of 463 K.

At the beginning of our experiment, we study the relation between electron yield and ion current. Total electron yield *γ* as a function of ion current for 0.8 MeV Xe^17+^ impacting on SiC ceramics target at a temperature of 463 K is shown in Fig. 2. The indicated error bars show the statistical errors, which are calculated by Eq. (1) and error transfer formula. It shows that *γ* is constant at about 22.03 ± 0.21 e^−^/ion, within the range from 80 nA to 480 nA. So it can be concluded that *γ* is hardly affected by ion current, or the effect can be neglected in our experiment.

**Figure 3:**
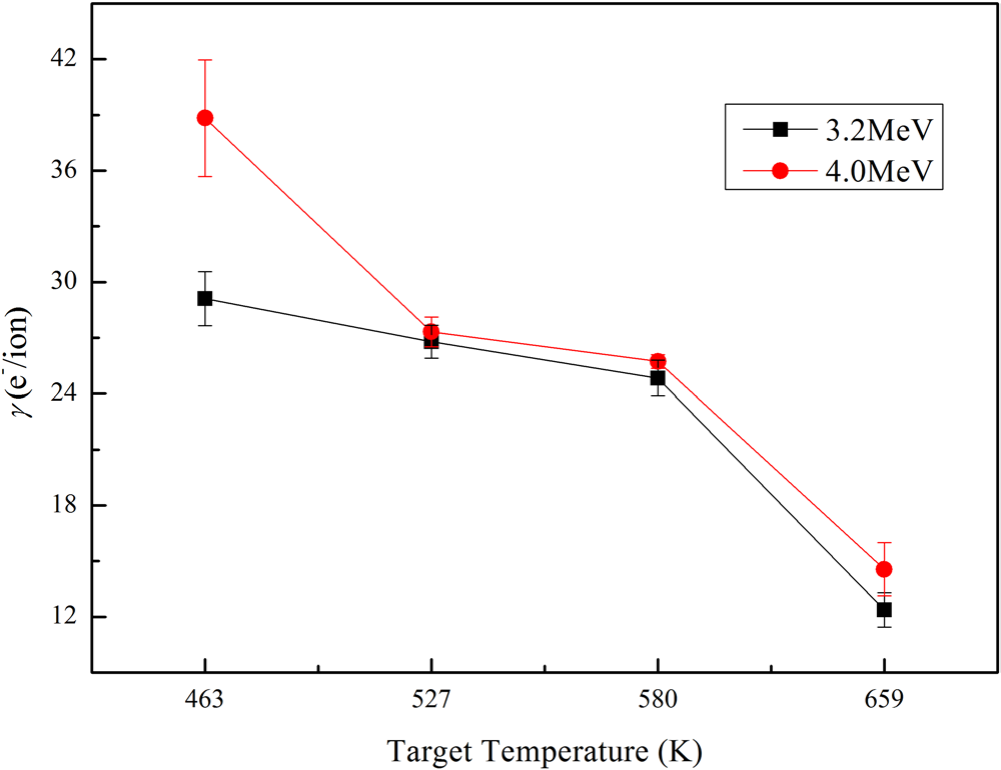
Total electron yield *γ* as a function of target temperature for 3.2 MeV and 4.0 MeV Xe^17+^ ions impacting on SiC ceramics in normal incident case.

Figure 3 represents total electron yield *γ* as a function of target temperature from SiC ceramics induced by Xe^17+^ in the normal incidence case. It shows that *γ* gradually decreases with increasing target temperature for 3.2 MeV and 4.0 MeV Xe^17+^ ions impacting on SiC target. The relative errors of these data are small, changing from 1.4% to 9.8%. We can conclude that target temperature can restrain secondary electron emission in our case. In the temperature range 463–659 K, the total decrease of *γ* is about 57% for 3.2 MeV Xe^17+^ impact, and about 62% for 4.0 MeV Xe^17+^ impact. This effect is much larger than the results observed for light ion impact and impact of heavy ions at low charged state^13–17^. Here we will discuss our results in terms of work function, because the work function of target, which determines the threshold energy, may depend on temperature and could be responsible for the observed temperature effect. The temperature dependence of work function has not been studied extensively until now^24^. Through others’ study^24–26^, we can find that work function increases with temperature increasing.

Both kinetic emission process (KE) and potential emission process (PE) can be influenced by the change of work function. KE is due to the excitation of target electrons by transfer of kinetic energy from incident ions. In PE, electrons are liberated in front of the surface by resonance or Auger neutralization of incident ions. For highly charged ions impacting, the total yield *γ* is divided into two parts, one is kinetic electron yield *γ*_*KE*_, and the other is potential electron yield *γ*_PE_. *γ*_KE_ almost vanishes when incident velocities *v* below “classical” kinetic emission threshold velocity *v*_th_^18, 19^, which is given by

2 $${\gamma }_{{\rm{KE}}}=k({v}-{{v}}_{{\rm{th}}}){\rm{\Theta }}({v}-{{v}}_{{\rm{th}}})$$

where Θ(*v* − *v*_th_) is a step function starting at the KE threshold velocity *v*_th_. According to Baragiola *et al*.^27^,

3 $${{v}}_{{\rm{th}}}=\frac{{{v}}_{F}}{2}[{(1+{W}_{\varphi }/{E}_{F})}^{1/2}-1]$$

where *W*_*φ*_ is work function, *E*_*F*_ is the Fermi-energy, *v*_*F*_ is the Fermi-velocity. When *v*å *v*_th_, there is an approximately linear relationship between *γ*_KE_ and *v*_th_. From Eqs. (2) and (3), we can conclude that *γ*_KE_ will decrease with increasing *W*_*φ*_.

Concerning the PE process now, all these processes require a minimum potential energy of at least twice the binding energy *W*_*φ*_ of the highest occupied state of the solid^28^. The maximum possible number of electrons ${n}_{\max }$ emitted via PE is therefore given by

4 $${n}_{\max }={E}_{pot}/2{W}_{\varphi }$$

where *W*_*φ*_ is the binding energy of the highest occupied state of the solid (which in the case of metal targets corresponds to the work function), *E*_pot_ is the potential energy of projectile, the potential energy of Xe^17+^ is about 2996 eV. But the maximum cannot be realized, and only a small portion of the potential energy is utilized in PE, because a portion of the potential excited electrons should not escape from the surface^29^. The classical *γ*_PE_ is concluded as

5 $${\gamma }_{{\rm{PE}}}\approx \frac{0.2}{{E}_{{\rm{F}}}}(0.8{E}_{{\rm{pot}}}-2{W}_{\varphi })$$

where *E*_F_ is Fermi energy of the target material. From Eq. (5), we can find that *γ*_PE_ decreases with increasing *W*_*φ*_. So, both *γ*_*KE*_ and *γ*_PE_ decreases with increasing *W*_*φ*_, that is to say, they decrease with increasing temperature, which corresponds to our experimental results.

**Figure 4:**
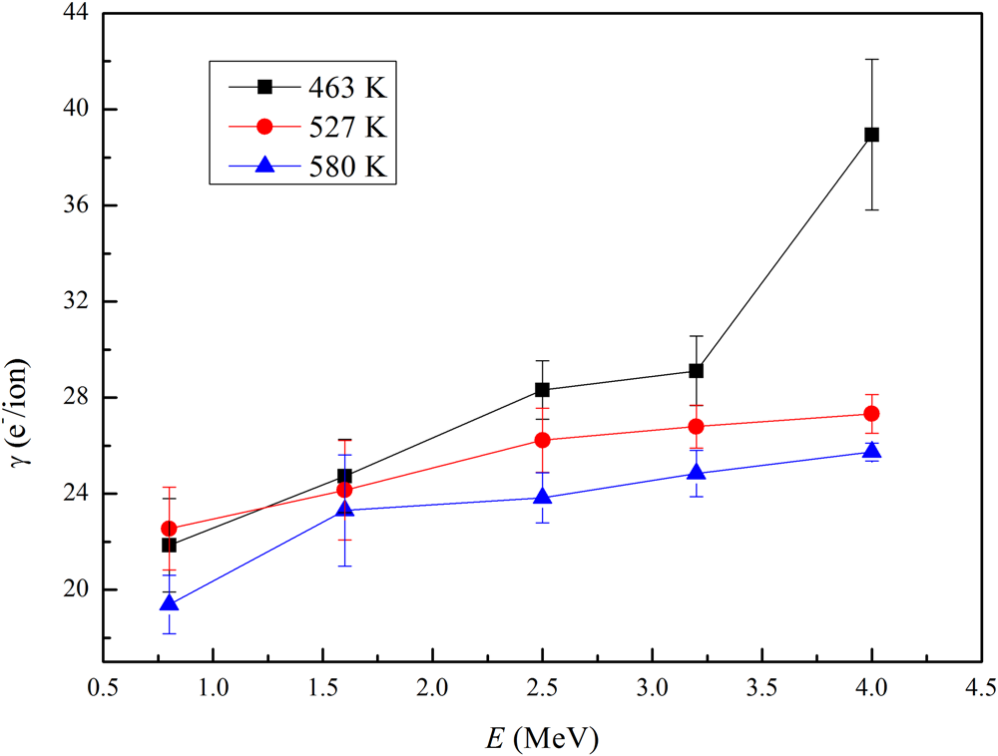
Total electron yield as a function of projectile energy for Xe^17+^ impacting on SiC ceramics at different target temperatures at normal incident case.

Total electron yield as a function of projectile energy for Xe^17+^ impacting on SiC ceramics surface at different target temperatures is shown in Fig. 4. The result shows that the total electron yield *γ* gradually increases with projectile energy, where *γ* also includes *γ*_*KE*_ and *γ*_*PE*_. From Eq. (5), we can know that *γ*_*PE*_ is a constant here, when the target is at a constant high temperature^30^. And *γ*_*KE*_ is usually given by

6 $${\gamma }_{KE}=\frac{B{S}_{e}}{\sin (\Psi )}$$

where *Se* is the electronic stopping power, Ψ is the incident angle of projectile relative to the surface normal, and *B* is nearly a constant factor which decreases slightly with increasing kinetic energy and atomic number of projectile^31^. In our experiment, the ion beam was at normal incident case, that is to say,$\sin (\Psi )=1$, so

7 $${\gamma }_{({Se})}=B{S}_{e}$$

**Figure 5:**
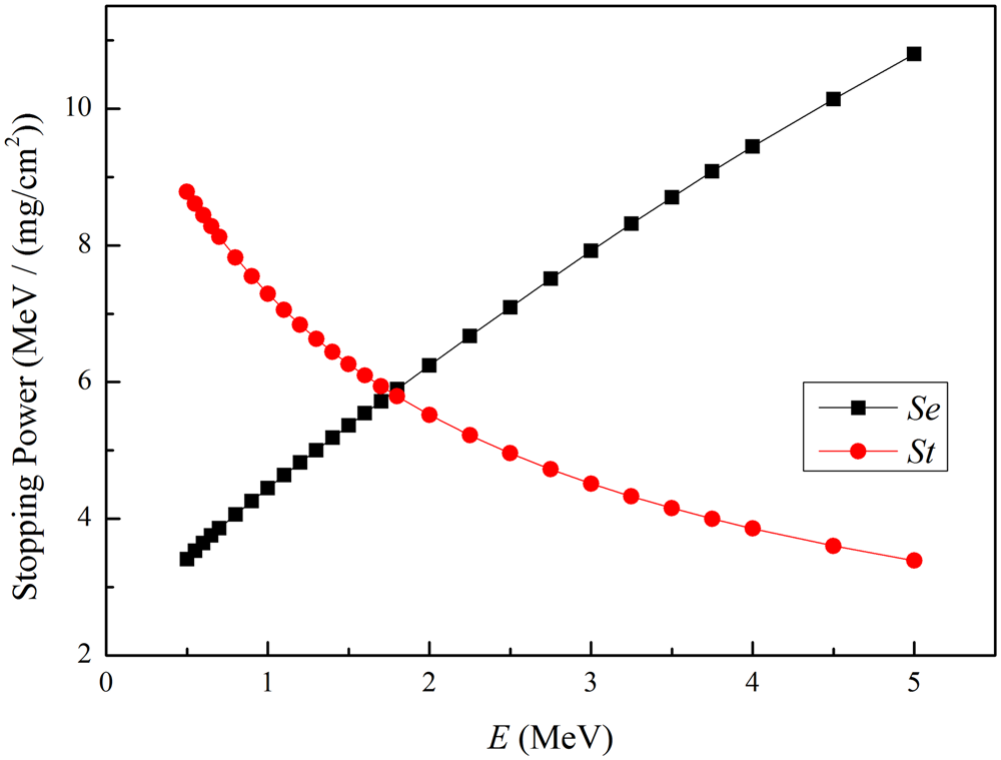
Electronic stopping power *Se* and nuclear stopping power *St* as a function of projectile energy for Xe^17+^impacting on SiC ceramics calculated by SRIM2008.

As shown in Fig. 5, the electronic stopping power *Se* and the nuclear stopping power *St* are functions of projectile energy for Xe^17+^ impacting on SiC ceramics surface calculated by SRIM2008. From Figs 4 and 5, we can conclude that the electron emission yield*γ* increases with the electronic stopping power *Se* of Xe^17+^ for high target temperature, which is consistent with the results at room temperature. Here the electron emission due to recoil atoms caused by the nuclear stopping power *St* can be neglected. The nuclear stopping power mainly contributes to sputtering ion yield.

The probably most important effect is the temperature dependence of the Fermi distribution of target electrons, which, for sufficiently high temperatures, can even result in thermal emission. However, it is noteworthy that this should increase the yield with increasing temperature, in contrast to our measurements. It is found that the change of the work function contributes significantly to the change of yield, but it may not be the only cause for the observed change of the yield. Further studies are in progress.

## Conclusions

Secondary electron emission yield from the surface of SiC ceramics has been studied induced by Xe^17+^ ions. We found that increasing target temperatures can be employed to decrease the electron emission yield induced by highly charged ions. The decrease is much larger than the decrease for single or low charged ions impact, that is to say, the temperature effect on electron emission is obvious for highly charged ions impact. We explain the results in terms of work function, by which both kinetic electron emission and potential electron emission can be influenced. Meanwhile, we also find the electron emission yield depends on projectile energy at a constant target temperature. Our research will supply useful data to the studies about temperature effect on secondary electron emission and the surface analysis of SiC ceramics.
